# First report of *Toxoplasma gondii* infection in market-sold adult chickens, ducks and pigeons in northwest China

**DOI:** 10.1186/1756-3305-5-110

**Published:** 2012-06-07

**Authors:** Wei Cong, Si-Yang Huang, Dong-Hui Zhou, Min-Jun Xu, Song-Ming Wu, Chao Yan, Quan Zhao, Hui-Qun Song, Xing-Quan Zhu

**Affiliations:** 1 State Key Laboratory of Veterinary Etiological Biology, Key Laboratory of Veterinary Parasitology of Gansu Province, Lanzhou Veterinary Research Institute, Chinese Academy of Agricultural Sciences, Lanzhou, Gansu Province, 730046, People’s Republic of China; 2 College of Animal Science and Technology, Jilin Agriculture University, Changchun, Jilin Province, 130118, People’s Republic of China; 3 Department of Pathogen Biology and Immunology, Laboratory of Infection and Immunity, Xuzhou Medical College, Xuzhou, Jiangsu Province, 221004, People’s Republic of China; 4 College of Animal Science and Technology, Yunnan Agricultural University, Kunming, Yunnan Province, 650201, People’s Republic of China

## Abstract

**Background:**

*Toxoplasma gondii* infection is a global concern, affecting a wide range of warm-blooded animals and humans worldwide, including poultry. Domestic and companion birds are considered to play an important role in the transmission of *T. gondii* to humans and other animals. However, little information on *T. gondii* infection in domestic birds in Lanzhou, northwest China was available. Therefore, this study was performed to determine the seroprevalence of *T. gondii* infection in domestic birds in Lanzhou, northwest China.

**Methods:**

In the present study, the seroprevalence of *T. gondii* antibodies in 413 (305 caged and 108 free-range) adult chickens, 334 (111 caged and 223 free-range) adult ducks and 312 adult pigeons in Lanzhou, northwest China, were examined using the modified agglutination test (MAT).

**Results:**

30 (7.26%) chickens, 38 (11.38%) ducks and 37 (11.86%) pigeons were found to be positive for *T. gondii* antibodies at the cut-off of 1:5. The prevalences in caged and free-range chickens were 6.23% and 10.19% respectively, however, statistical analysis showed that the difference was not significant (*P >* 0.05). The seroprevalences in caged and free-range ducks were 6.31% and 13.90% respectively, but the difference was not statistically significant (*P >* 0.05).

**Conclusions:**

The results of the present survey indicated the presence of *T. gondii* infection in adult chickens, ducks and pigeons sold for meat in poultry markets in Lanzhou, northwest China, which poses a potential risk for *T. gondii* infection in humans and other animals in this region. This is the first seroprevalence study of *T. gondii* infection in domestic birds in this region.

## Background

*Toxoplasma gondii* is an important intracellular protozoan parasite, widely prevalent in humans and animals, including poultry throughout the world [1-3]. *T. gondii* infection is generally transmitted either congenitally, or via ingestion of undercooked or raw meat from infected animals, or ingestion of food or water contaminated with oocysts excreted by infected felids [1,2,4]. Free-range (FR) chickens, an important intermediate host, are considered one of the best indicators for soil contamination with *T. gondii* oocysts because of their feeding style [2]. Low numbers of exposed poultry develop clinical symptoms, such as encephalitis, chorioretinitis and neuritis, however, poultry meat is an important part of cuisine, consumed widely all over the world, and consumption of uncooked poultry meat or not properly cooked poultry meat is a risk factor for *T. gondii* infection in humans or other animals [1,2].

In recent years, seroprevalence studies of *T. gondii* in chickens, ducks and pigeons have been conducted extensively in various parts of the world [1,2,5-7], there have been some surveys in mainland China [8-10]. However, little is known about the prevalence of *T. gondii* in chickens, ducks and pigeons in northwest China. Here, we report *T. gondii* seroprevalence in domestic birds in Lanzhou, northwest China for the first time.

## Methods

### The study area

The survey was conducted in Lanzhou City (35°5′~38°N, 102°30′~104°30′ E), the Capital of Gansu Province, northwest China. Lanzhou is situated in the geometric center of China, covering an area of approximately 13,000 square kilometers, with an average altitude of 1,500 meters. The climate of this city is typically temperate and monsoonal continental, with an average annual temperature of 9.3 °C, and an annual precipitation of 360 mm.

### Blood samples

A total of 413 blood samples from adult chickens, 334 blood samples from adult ducks and 312 blood samples from adult pigeons were collected from animals slaughtered and sold for meat in four poultry markets (poultry market A, B, C, D) in Lanzhou, northwest China between April and November 2011. Blood samples were transported to the laboratory in Lanzhou Veterinary Research Institute, Chinese Academy of Agricultural Sciences, Lanzhou, Gansu Province, China, kept at room temperature for 2 hr, centrifuged at 3,000 *g* for 10 min, then clear serum was separated. The serum samples obtained were stored at −20 °C until further analyzed. Owners of poultry were asked for information of animal husbandry practices.

### Serological examination

Antibodies to *T. gondii* were determined in chicken, duck and pigeon sera by the modified agglutination test (MAT) as described previously [2,8,11,12]. In brief, sera were added to the “U” bottom of 96 well microtiter plates, and diluted two-fold starting from 1:5 to 1:160. Bird sera with MAT titers of 1:5 or higher were considered positive for *T. gondii* antibodies based on previous studies [2,8,13-15], those sera with doubtful reactions were re-tested, and positive and negative controls were included in each test.

### Statistical analyses

Differences in the seroprevalence of *T. gondii-*infected chickens and ducks and between free-range and caged groups were analyzed using a Chi square test in SPSS for Windows (Release 18.0 standard version, SPSS Inc., Chicago, Illinois). The differences were considered statistically significant when *P* < 0.05.

## Results

One hundred and five (9.92%) out of 1059 serum samples were assayed positive for *T. gondii* by MAT (Table 1). Of these, a total of 30 (7.26%) out of 413 chickens were seropositive and antibody titers were 1:5 in 20, 1:10 in seven, 1:20 in one, 1:40 in one and 1:80 in one chicken (Table 1). As shown in Table 2, the seroprevalence varied in different poultry markets, ranging from 1.73% to 10.19%, and the seroprevalence in caged and free-range chickens was 6.23% and 10.19%, respectively.

**Table 1:** **Seroprevalence of*Toxoplasma gondii*infection in chickens, ducks and pigeons in Lanzhou, northwest China by modified agglutination test (MAT)**

**Host**	**No. of sera with MAT titers of:**					**No. positive**	**No. tested**	**Prevalence (%)**
	**1:5**	**1:10**	**1:20**	**1:40**	**1:80**			
Caged chickens	13	4	0	1	1	19	305	6.23
FR chickens	7	3	1	0	0	11	108	10.19
Caged ducks	7	0	0	0	0	7	111	6.31
FR ducks	26	5	0	0	0	31	223	13.90
Pigeons	35	2	0	0	0	37	312	11.86
Total	88	14	1	1	1	105	1059	9.92

FR: Free-range.

**Table 2:** **Seroprevalence of*Toxoplasma gondii*infection in chickens, ducks and pigeons in different poultry markets in Lanzhou, northwest China by modified agglutination test (MAT)**

**Host**	**Poultry market**	**No. tested**	**No. positive**	**Prevalence (%)**
Chicken	A	97	9	9.28
	B	113	2	1.77
	C	108	11	10.19
	D	95	8	8.42
Duck	A	63	4	6.35
	B	43	0	0
	C	62	4	6.45
	D	166	30	18.07
Pigeon	A	51	3	5.88
	B	20	3	15
	C	119	3	2.52
	D	122	28	22.95

Antibodies to *T. gondii* were found in 38 (11.38%) out of 334 ducks, and antibody titers were 1:5 in 33 and 1:10 in five ducks (Table 1). *T. gondii* seroprevalences in ducks came from four different poultry markets ranging from 0% to 18.07% (Table 2). The investigation revealed that the prevalence in caged and free-range ducks was 17.61% and 12.72%, respectively.

*T. gondii* antibodies were detected in 37 (11.86%) of 312 examined pigeons with antibody titers of 1:5 in 35 and 1:10 in two (Table 1), and the seroprevalences of *T. gondii* in four different poultry markets ranged from 2.52% to 22.95% (Table 2).

## Discussion

In this investigation, seroprevalence of *T. gondii* infection in chickens was 7.26%, which was lower than that observed in other countries [2], and also lower than that reported in Jingzhou city (25.17%) [10], but similar to that in Zhangjiakou city (7.41%) [9], and Guangzhou city (8.43%) [8]. The seroprevalence in caged chickens and free-range chickens was 6.23% and 10.19%, respectively, although the difference was not statistically significant (*P >* 0.05).

The overall *T. gondii* seroprevalence in ducks in Lanzhou was 11.38%, which was lower than that reported in other countries [16-18], and also lower than that reported in Guangzhou city (16%) [8]. The differences in seroprevalence may due to differences in ecological and geographical factors. Of these, the *T. gondii* seroprevalence in caged ducks and free-range ducks were 6.31% and 13.90%, respectively, but the difference was not statistically significant (*P >* 0.05), probably related to different life styles of the examined ducks.

To our knowledge, there was only one report regarding toxoplasmosis in pigeons in mainland China [14]. In the present study, antibodies to *T. gondii* were found in 37 (11.86%) pigeons at the cut-off of 1:5, but most had low titres, with MAT titres of 1:5 in 35 pigeons, only 2 pigeons had titres of 1:10 or higher. This seroprevalence was higher than that in Guangdong Province (8.7%) [14], possibly indicating geographical differences. Pigeon meat can serve as a source of *T. gondii* infection for hunters and other animals, so it would be a risk factor for *T. gondii* infection in humans or other animals.

In this study, we chose MAT because it is sensitive and specific for detecting *T. gondii* antibodies in bird species [2,8,13-15], compared to other serologic methods. Moreover, geographical conditions, feeding and living styles, and number of cats and rodents may contribute to the differences in *T. gondii* seropositivity in birds.

## Conclusions

The present survey revealed the seroprevalence of *T. gondii* in chickens, ducks and pigeons in Lanzhou, northwest China for the first time, which indicated the potential risk of domestic birds as a source of *T. gondii* infection in humans and other animals in this region.

## Competing interests

The authors declare that they have no competing interests.

## Authors' contributions

XQZ conceived and designed the study, and critically revised the manuscript. WC, SYH, DHZ, MJX and SMW performed the experiments, analysed the data and drafted the manuscript. CY, QZ, and HQS helped in study design, study implementation and manuscript revision. All authors read and approved the final manuscript.
